# Editorial: Plant-Pest Interactions Volume III: Coleoptera and Lepidoptera

**DOI:** 10.3389/fpls.2021.730290

**Published:** 2021-08-11

**Authors:** Takeshi Suzuki, George Broufas, Guy Smagghe, Félix Ortego, Colette Broekgaarden, Isabel Diaz

**Affiliations:** ^1^Graduate School of Bio-Applications and Systems Engineering, Tokyo University of Agriculture and Technology, Koganei, Japan; ^2^Department of Agricultural Development, Faculty of Agricultural Sciences and Forestry, Democritus University of Thrace, Orestiada, Greece; ^3^Department of Plants and Crops, Ghent University, Ghent, Belgium; ^4^Centro de Investigaciones Biologicas Margarita Salas, CSIC, Madrid, Spain; ^5^KeyGene N.V. Agro Business Park 90, Wageningen, Netherlands; ^6^Centro de Biotecnología y Genómica de Plantas, Universidad Politécnica de Madrid (UPM)-Instituto Nacional de Investigación y Tecnología Agraria y Alimentaria, Madrid, Spain; ^7^Departamento de Biotecnología-Biología Vegetal, Escuela Técnica Superior de Ingeniería Agronómica, Alimentaria y de Biosistemas, UPM, Madrid, Spain

**Keywords:** lepidoptera, coleoptera, plant defences, resistance, multiple interactions

The study of plant-pest interactions is a fast-moving research field built around the defence/counter-defence interchange between adversaries. The survival within this battle requires a high metabolic cost for both partners who, as a result of millions of years of coexistence, have developed weapons against each other. Progress, particularly on the molecular analyses of this relationship has been published in the last years, revealing a specific gene reprogramming dependent on the interactor species. Plant-pest interactions have been found to be associated with a battery of key elements, metabolic pathways, regulators and defensive metabolites, as well as physical barriers and behavioural changes (reviewed by Santamaria et al., 2018; Stahl et al., 2018; Erb and Reymond, 2019; Wilkinson et al., 2019; Hamann et al., 2021).

This Research Topic is addressed in a special issue on plant-pest interactions which has been divided in three volumes based on the pest order. This volume III is focussed on coleopteran and lepidopteran species.

The two orders with the greatest number of plant-feeding species, mainly biting-chewing beetles and caterpillars, have a high impact in agriculture since they consume large portions of plant tissues. Plants recognise this damage together with the herbivore-associated molecular patterns (HAMPs), and activate downstream responses triggering hormonal-regulated direct and indirect defences. In turn, biting-chewing beetles and caterpillars have evolved strategies to overcome these defences (Basu et al., 2018; Stahl et al., 2018). In this context, the four articles included in volume III present important and novel perspectives to the subject.

Chen et al. have investigated the differential molecular mechanisms underlying cotton plant defences against the bollworm *Helicoverpa armigera* and the mirid bug *Apolygus lucorum*, two pests with different feeding habits. They describe, at transcriptional level, how genes involved in defence signalling, hormonal regulation and final defensive products, are differentially expressed in cotton cotyledons depending on the feeder. The most important result deals with the mechanism of alternative splicing by which one gene may produce multiple different transcripts and in consequence, generate different proteins (Yang et al., 2014). Interestingly, the present work describes how the alternative splicing patterns differ in cotton in response to the two insect infestations, indicating that this co-transcriptional regulatory mechanism is also required for defences against pests.

An important aspect of the plant-pest interaction is the plant response to eggs laid by the herbivore, to particularly know whether oviposition mediates plant priming defences against hatching larvae or suppress them. Some nice publications have reported both antagonistic effects, mainly in lepidopteran species (Bruessow et al., 2010; Hilfiker et al., 2014). In this case, Valsamakis et al. have analysed how long the eggs from the cabbage butterfly *Pieris brassicae* need to remain on Arabidopsis plants to prime defences. Results show that larvae gain less biomass the longer the eggs have been on the plant, making to be the time coincident with *P. brassicae* embryo development inside the egg. Hence, it looks that the plant is preparing its defences just in time prior to larval hatching.

Plants and pests search their ecological niches with other organisms and the combination of biotic and abiotic factors may alter their behaviour and physiology. In this scenario, Chalivendra et al. had observed in field trials, a preference in the natural infestation of the corn earworm *Helicoverpa zea* to specific maize genotypes with contrasting levels of resistance to *Aspergillus flavus* that correlated with seed fumonisin contamination by native *Fusarium verticillioides* strains. Since mycotoxins are very relevant for food safety, they have studied the factors underlying the host-pathogen-insect interaction and found that the host genotype even with demonstrable resistance can become vulnerable due to variation in flowering time and the outbreak of chewing insects. They could conclude that the incorporation of resistance to a single micotoxin accumulation not always pairs with insect resistance.

Regarding this multifactorial interaction between organisms, an article by Wang et al. has shown the profiles of volatile organic compounds (FVOCs) emitted by two ophiostomatoid fungi (*Grosmannia clavigera* and *Ophiostoma ips*) associated with two species of pine beetles and how can be influenced by the FVOC emissions from other ophiostomatoid fungi. The results suggest that the similarities in fungal volatiles may reflect a common ecological niche while differences may correspond to species-specific adaptation to their respective hosts or genetic factors.

The information reported in this volume III on plant-pest interaction, has added key elements in plant-coleopteran/lepidopteran insect interplay, but further research is needed to get a full understanding and for exploiting natural defence mechanism in agriculture.

## Author Contributions

All authors have participated in the article writing and have acted as coeditors of this special issue.

## Conflict of Interest

The authors declare that the research was conducted in the absence of any commercial or financial relationships that could be construed as a potential conflict of interest.

## Publisher's Note

All claims expressed in this article are solely those of the authors and do not necessarily represent those of their affiliated organizations, or those of the publisher, the editors and the reviewers. Any product that may be evaluated in this article, or claim that may be made by its manufacturer, is not guaranteed or endorsed by the publisher.
